# Household Ventilation May Reduce Effects of Indoor Air Pollutants for Prevention of Lung Cancer: A Case-Control Study in a Chinese Population

**DOI:** 10.1371/journal.pone.0102685

**Published:** 2014-07-14

**Authors:** Zi-Yi Jin, Ming Wu, Ren-Qiang Han, Xiao-Feng Zhang, Xu-Shan Wang, Ai-Ming Liu, Jin-Yi Zhou, Qing-Yi Lu, Claire H. Kim, Lina Mu, Zuo-Feng Zhang, Jin-Kou Zhao

**Affiliations:** 1 Department of Non-communicable Chronic Disease Control, Jiangsu Provincial Center for Disease Control and Prevention, Nanjing, Jiangsu, China; 2 Jiangyin Center for Disease Control and Prevention, Jiangyin, Jiangsu, China; 3 Ganyu Center for Disease Control and Prevention, Ganyu, Jiangsu, China; 4 Dafeng Center for Disease Control and Prevention, Dafeng, Jiangsu, China; 5 Center for Human Nutrition, David Geffen School of Medicine, University of California Los Angeles (UCLA), Los Angeles, California, United States of America; 6 Department of Epidemiology, Fielding School of Public Health, University of California Los Angeles (UCLA), Los Angeles, California, United States of America; 7 Department of Social and Preventive Medicine, State University of New York at Buffalo, Buffalo, New York, United States of America; MOE Key Laboratory of Environment and Health, School of Public Health, Tongji Medical College, Huazhong University of Science and Technology, China

## Abstract

**Background:**

Although the International Agency for Research on Cancer (IARC) has classified various indoor air pollutants as carcinogenic to humans, few studies evaluated the role of household ventilation in reducing the impact of indoor air pollutants on lung cancer risk.

**Objectives:**

To explore the association between household ventilation and lung cancer.

**Methods:**

A population-based case-control study was conducted in a Chinese population from 2003 to 2010. Epidemiologic and household ventilation data were collected using a standardized questionnaire. Unconditional logistic regression was employed to estimate adjusted odds ratios (OR_adj_) and their 95% confidence intervals (CI).

**Results:**

Among 1,424 lung cancer cases and 4,543 healthy controls, inverse associations were observed for good ventilation in the kitchen (OR_adj_ = 0.86, 95% CI: 0.75, 0.98), bedroom (OR_adj_ = 0.90, 95% CI: 0.79, 1.03), and both kitchen and bedroom (OR_adj_ = 0.87, 95% CI: 0.75, 1.00). Stratified analyses showed lung cancer inversely associated with good ventilation among active smokers (OR_adj_ = 0.85, 95% CI: 0.72, 1.00), secondhand smokers at home (OR_adj_ = 0.77, 95% CI: 0.63, 0.94), and those exposed to high-temperature cooking oil fumes (OR_adj_ = 0.82, 95% CI: 0.68, 0.99). Additive interactions were found between household ventilation and secondhand smoke at home as well as number of household pollutant sources.

**Conclusions:**

A protective association was observed between good ventilation of households and lung cancer, most likely through the reduction of exposure to indoor air pollutants, indicating ventilation may serve as one of the preventive measures for lung cancer, in addition to tobacco cessation.

## Introduction

Lung cancer is the leading cause of cancer death in males and the second leading cause of cancer death in females in the world. It accounted for 18.2% of all cancer deaths worldwide in 2008, with an age-standardized mortality rate (ASR) of 19.4 per 100,000 [1], [2]. Lung cancer was also the most common cause of cancer death in China, leading to 452,813 deaths and an ASR of 28.7 per 100,000 in 2008 [3], [4].

Risk factors associated with lung cancer have been examined extensively. Sufficient evidence confirmed tobacco smoking (including involuntary or secondhand smoke) is the most critical risk factor for lung cancer [5], [6]. Family history of lung cancer and occupational exposure to carcinogens such as asbestos, arsenic and crystalline silica are also established risk factors for lung cancer [7]. A number of indoor air pollutants, such as emissions from household combustion of coal, coal gasification, coke production, and radon-222 and its decay products, have been classified by the International Agency for Research on Cancer (IARC) as lung carcinogens with sufficient evidence in humans [7]. In addition, indoor emissions from household combustion of biomass fuel (primarily wood), and fumes from high-temperature cooking oil have been classified as probably carcinogenic to humans (Group 2A) by the IARC [8]. In China, indoor air pollutants are comprised of secondhand smoke, emissions from solid fuel used for heating and cooking, and cooking oil fumes [9].

Adequate ventilation in the household would reduce the exposure to indoor air pollutants, which in turn will reduce the risk of lung cancer for people who lived in the household. However, only a few studies have examined the protective role of ventilation on lung cancer. A previous retrospective cohort study reported that reduced lung cancer mortality is associated with changing from unvented stoves to portable stoves or stoves with chimneys in both male and female lifetime smoky coal users in Xuanwei, China [10], [11]. Two case-control studies of Chinese population reported inverse associations between good ventilation and lung cancer in Guangzhou, Southern China and Taiyuan, Northern China [12], [13]. However, those studies did not examine the potential effect modification between ventilation and indoor air pollution.

To evaluate the role of household ventilation in the kitchen and bedroom and the potential lung cancer risk reduction due to indoor air pollution, we conducted a population-based case–control study with a large sample in two counties of Jiangsu Province, China.

## Materials and Methods

### Study design, participants and data collection

Details of this study have been described in a previous study, which suggested protective association between consumption of raw garlic and lung cancer [14]. In brief, a population-based case-control study was conducted in Dafeng and Ganyu counties in Jiangsu Province, China, from 2003 to 2010. Both counties are economically under-developed, rural areas in the coastal region of northern Jiangsu. Dafeng and Ganyu have approximately 0.7 million and 1.1 million inhabitants respectively. Agricultural population accounts for 64.8% of total local residents in Dafeng and 55.1% in Ganyu. The two counties have very similar lung cancer mortality, with an average of 20.5 per 100,000 from 1996 to 2002 [15].

Eligible cases were newly diagnosed primary lung cancer patients identified from the local population-based tumor registries. Eligible controls were randomly selected from each county’s demographic database, matched with cases on gender and age (±5 years). A total of 1,424 cases (625 in Dafeng and 799 in Ganyu) and 4,543 controls (2,533 in Dafeng and 2,010 in Ganyu) were included in this study. Participation rates were 39.5% and 56.8% for cases and 87% and 85% for controls in Dafeng and Ganyu, respectively.

The Institutional Review Board of Jiangsu Provincial Health Department approved the protocol of this study. Participants read and signed the informed consent form prior to entering the study. Using a standardized epidemiological questionnaire [16], trained interviewers collected data on basic demographic factors, socioeconomic status, tobacco smoking history, alcohol consumption, family history of cancers, dietary history, and physical activity. In addition, particular efforts were made to collect data on exposure to household indoor air pollutants, such as secondhand smoke at home, high-temperature cooking oil fumes, cooking fuels (kerosene, coal, natural gas, liquefied gas, coal gas, electricity, firewood and straw), and heating fuels (coal stove, firewood and straw, coke, ondol heating, central heating and electricity). Information regarding ventilation conditions in both the kitchen and the bedroom were also collected through the questionnaire. Ventilation quality was categorized into good, fair, or poor, based on interviewers’ observation of household conditions such as the number and size of windows and participants’ self-report on usual ventilation. For good, fair and poor ventilation of a house, the average number of windows were 7, 4 and 3, respectively, and the average total size of windows were 10, 6 and 4 square meters, respectively.

### Statistical analysis

Firewood and straw were universally used for cooking (91.2% among cases and 93.5% among controls), so they were not considered in the analysis. The other types of cooking fuel were dichotomized into coal (including kerosene) versus others (natural gas, liquefied gas, coal gas, and electricity). Heating fuel was dichotomized into solid fuels (coal stove, firewood and straw, coke and ondol heating) versus others (central heating, electricity, and no heating). Because the proportions of individuals living in households with poorly ventilated kitchens or bedrooms were very low (7.7% and 7.3%, respectively), we combined the categories of “poor” and “fair.” Thus, kitchen ventilation and bedroom ventilation were dichotomized into good versus not good. Overall ventilation quality in the household was classified into three categories: good (good ventilation in both the kitchen and the bedroom), fair (good ventilation in either the kitchen or the bedroom) and poor (good ventilation in neither the kitchen nor the bedroom). In the analysis of overall household ventilation in reducing exposure to indoor air pollutants, high-temperature cooking oil and coal used for cooking were included as pollutant sources in the kitchen, solid fuels used for heating was included as a pollutant source in the bedroom, and tobacco smoke from active smoking and secondhand smoke at home were considered pollutant sources in both places. The distributions of basic characteristics of cases and controls were compared using the Chi-squared test. Unconditional logistic regression was used to estimate odds ratios (ORs) and their 95% confidence intervals (CIs) for the associations between variables related to indoor air pollution and lung cancer. Joint effects of household ventilation and indoor air pollutants were evaluated. For multiplicative interaction, ratio of ORs (ROR) was generated by including main effect variables and their product terms in a logistic regression model. Additive interaction was examined based on three measures – the relative excess risk due to interaction (RERI), attributable proportion due to interaction (AP), and synergy index (S) [17]–[19]. The stratum with the lowest risk served as the reference category [20]. RERI and AP are equal to 0 and S is equal to 1 in the absence of additive interaction. Multivariate models were adjusted for potential confounders, which were selected based on prior knowledge and confounding assessment; these included age (continuous), gender, education level (illiterate/primary school/middle school/high school/college), income 10 years ago (Yuan/year, continuous), body mass index (BMI, kg/m^2^, continuous), family history of lung cancer (yes/no), pack-years of smoking (continuous), ethanol consumption (ml/week, continuous) and study area (Dafeng/Ganyu). We also examined the associations after stratifying the data by county, ever smoking and gender. Epidata 3.0 (EpiData Association, Denmark) was used for data entry and SAS v9.2 (SAS Institute, Inc., Cary, NC) was used for data cleaning and analysis.

## Results

Table 1 shows the characteristics of cases and controls. Among both males and females, cases were more likely than controls to be underweight (BMI <18.5 kg/m^2^) and to smoke cigarettes. Among males, cases were more likely than controls to have an education level of primary school or above, have a family history of lung cancer, and to consume alcohol. Among females, cases tended to be younger and less likely to consume alcohol than controls.

**Table 1:** Characteristics of cases and controls.

Variables	Male		*P-*value a	Female		*P-*value a
	Control (%) (N = 3,415)	Case (%) (N = 995)		Control (%) (N = 1,128)	Case (%) (N = 429)	
**Study area**						
Dafeng	1,815 (53.1)	442 (44.4)		718 (63.7)	183 (42.7)	
Ganyu	1,600 (46.9)	553 (55.6)	<0.001	410 (36.3)	246 (57.3)	<0.001
**Age (year)**						
<50	374 (11.0)	106 (10.7)		125 (11.1)	60 (14.0)	
50–	783 (22.9)	233 (23.4)		206 (18.3)	95 (22.1)	
60–	1,080 (31.6)	330 (33.2)		360 (31.9)	138 (32.2)	
70–	962 (28.2)	268 (26.9)		347 (30.8)	119 (27.7)	
≥80	216 (6.3)	58 (5.8)	0.837	90 (8.0)	17 (4.0)	0.012
**Education level**						
Illiteracy	1,441 (42.2)	370 (37.2)		868 (77.0)	336 (78.3)	
Primary	1,217 (35.6)	399 (40.1)		176 (15.6)	67 (15.6)	
Middle & above	757 (22.2)	226 (22.7)	0.011	84 (7.4)	26 (6.1)	0.631
**Income 10 years ago (Yuan/year)**						
<1000	734 (21.5)	202 (20.3)		225 (19.9)	82 (19.1)	
1000–	613 (18.0)	180 (18.1)		230 (20.4)	86 (20.0)	
1500–	925 (27.1)	262 (26.3)		309 (27.4)	118 (27.5)	
≥2500	1,143 (33.5)	351 (35.3)	0.699	364 (32.3)	143 (33.3)	0.971
**BMI (kg/m^2^)** b						
<18.5	209 (6.1)	125 (12.6)		98 (8.7)	79 (18.4)	
18.5–23.9	2,236 (65.5)	630 (63.3)		645 (57.2)	232 (54.1)	
24–27.9	800 (23.4)	196 (19.7)		303 (26.9)	89 (20.7)	
≥28	170 (5.0)	44 (4.4)	<0.001	82 (7.3)	29 (6.8)	<0.001
**Family history of lung cancer**						
No	3,342 (97.9)	949 (95.4)		1,091 (96.7)	407 (94.9)	
Yes	73 (2.1)	46 (4.6)	<0.001	37 (3.3)	22 (5.1)	0.088
**Pack-years of smoking**						
Never smoker	1,013 (29.7)	100 (10.1)		847 (75.1)	294 (68.5)	
<30 years	908 (26.6)	196 (19.7)		180 (16.0)	66 (15.4)	
≥30 years	1,494 (43.7)	699 (70.3)	<0.001	101 (9.0)	69 (16.1)	<0.001
**Ethanol consumption**						
Never	1,036 (30.3)	257 (25.8)		901 (79.9)	352 (82.1)	
<500 ml/week	1,035 (30.3)	270 (27.1)		193 (17.1)	55 (12.8)	
≥500 ml/week	1,344 (39.4)	468 (47.0)	<0.001	34 (3.0)	22 (5.1)	0.022

a Based on Chi-squared tests.

b Chinese recommend standard was used for the cut-off points of overweight and obesity: underweight (BMI <18.5), normal weight (BMI 18.5–23.9), overweight (BMI 24.0–27.9), obese (BMI≥28.0).

The distributions of factors related to indoor air pollution and their associations with lung cancer are presented in Table 2. After adjusting for potential confounding factors, an inverse association was found between lung cancer and good ventilation in the kitchen (OR_adj = _0.86, 95% CI: 0.75, 0.98), and a borderline inverse association was observed for good ventilation in the bedroom (OR_adj_ = 0.90, 95% CI: 0.79, 1.03). Compared with poor ventilation in both the kitchen and the bedroom, good ventilation in both was inversely associated with lung cancer (OR_adj_ = 0.87, 95% CI: 0.75, 1.00). On the other hand, positive associations were observed between lung cancer and exposure to secondhand smoke at home (OR_adj_ = 1.41, 95% CI: 1.24, 1.60), high-temperature cooking oil vapor (OR_adj_ = 1.26, 95% CI: 1.10, 1.43), coal used for cooking (OR_adj_ = 1.38, 95% CI: 1.17, 1.62) and solid fuels used for heating (OR_adj_ = 1.27, 95% CI: 1.10, 1.47). When treating the cumulative number of pollutant sources as a continuous variable in logistic regression model, it was positively associated with lung cancer in a dose-response manner (OR_adj_ = 1.29, 95% CI: 1.21, 1.38) for each additional indoor pollution source. Compared with no pollutant source, OR for three or four pollutant sources and one or two pollutant sources was 1.97 (95% CI: 1.48, 2.63) and 3.01 (95% CI: 2.22, 4.08), respectively.

**Table 2:** Distribution of factors related to indoor air pollution and their associations with lung cancer risk.

Variables	Control (%) (N = 4,543)	Case (%) (N = 1,424)	Crude OR (95%CI)	Adjusted OR (95%CI)^a^
**Good ventilation in kitchen**				
No	2,618 (57.6)	887 (62.3)	1.00	1.00
Yes	1,925 (42.4)	537 (37.7)	0.82 (0.73,0.93)	0.86 (0.75,0.98)
**Good ventilation in bedroom**				
No	2,604 (57.3)	873 (61.3)	1.00	1.00
Yes	1,939 (42.7)	551 (38.7)	0.85 (0.75,0.96)	0.90 (0.79,1.03)
**Household ventilation**				
Poor	2,411 (53.1)	821 (57.7)	1.00	1.00
Fair	400 (8.8)	118 (8.3)	0.87 (0.70,1.08)	0.85 (0.68,1.07)
Good	1,732 (38.1)	485 (34.1)	0.82 (0.72,0.94)	0.87 (0.75,1.00)
**Secondhand smoke at home**				
No	2,742 (60.4)	688 (48.3)	1.00	1.00
Yes	1,801 (39.6)	736 (51.7)	1.63 (1.45,1.84)	1.41 (1.24,1.60)
**High-temperature cooking oil**				
No	3,194 (70.3)	911 (64.0)	1.00	1.00
Yes	1,349 (29.7)	513 (36.0)	1.33 (1.18,1.51)	1.26 (1.10,1.43)
**Coal used for cooking**				
No	3,317 (73.0)	868 (61.0)	1.00	1.00
Yes	1,226 (27.0)	556 (39.0)	1.73 (1.53,1.96)	1.38 (1.17,1.62)
**Solid fuels used for heating**				
No	1,238 (27.3)	348 (24.4)	1.00	1.00
Yes	3,305 (72.7)	1,076 (75.6)	1.16 (1.01,1.33)	1.27 (1.10,1.47)
**Number of pollutant sources**				
0	442 (9.7)	60 (4.2)	1.00	1.00
1 or 2	3,244 (71.4)	914 (64.2)	2.08 (1.57,2.75)	1.97 (1.48,2.63)
3 or 4	857 (18.9)	450 (31.6)	3.87 (2.89,5.18)	3.01 (2.22,4.08)
*P*_trend_			<0.001	<0.001
OR (continuous)			1.43 (1.34,1.52)	1.29 (1.21,1.38)

a Adjusted for age (continuous), gender, education level (illiterate/primary school/middle school/high school/college), income 10 years ago (Yuan/year, continuous), body mass index (kg/m^2^, continuous), family history of lung cancer (yes/no), pack-years of smoking (continuous), ethanol consumption (ml/week, continuous), and study area (Dafeng/Ganyu).

Table 3 shows the joint effects of household ventilation and indoor air pollutants on the risk of lung cancer. In the presence of each type of indoor air pollutant, good ventilation was inversely associated with lung cancer for ever smoking (OR_adj_ = 0.85, 95% CI: 0.72, 1.00), secondhand smoke at home (OR_adj_ = 0.77, 95% CI: 0.63, 0.94), high-temperature cooking oil fumes (OR_adj_ = 0.82, 95% CI: 0.68, 0.99), coal used for cooking (OR_adj_ = 0.89, 95% CI: 0.72, 1.11), and solid fuels used for heating (OR_adj_ = 0.94, 95% CI: 0.81, 1.10). There was some indication of additive interaction between household ventilation and secondhand smoke at home (RERI = −0.17, 95% CI: −0.35, 0.01; AP = −0.13, 95% CI: −0.26, 0.00; S = 0.67, 95% CI: 0.47, 0.95).

**Table 3:** Joint effects of household ventilation and indoor air pollutants on lung cancer risk.

Pollutant source	Household ventilation^a^	Control (%) (N = 4,543)	Case (%) (N = 1,424)	Crude OR (95%CI)	Adjusted OR (95%CI)^b^
**Ever smoking**					
Yes	Poor c	1,466 (32.3)	605 (42.5)	1.00	1.00
Yes	Fair	248 (5.5)	84 (5.9)	0.82 (0.63,1.07)	0.77 (0.59,1.01)
Yes	Good	969 (21.3)	341 (23.9)	0.85 (0.73,1.00)	0.85 (0.72,1.00)
No	Poor	945 (20.8)	216 (15.2)	0.55 (0.47,0.66)	0.39 (0.32,0.48)
No	Fair	152 (3.3)	34 (2.4)	0.54 (0.37,0.80)	0.37 (0.24,0.55)
No	Good d	763 (16.8)	144 (10.1)	0.46 (0.37,0.56)	0.33 (0.26,0.41)
Interaction b	Additive: RERI = −0.13 (−0.37,0.11); AP = −0.05 (−0.16,0.05);				
	S = 0.91 (0.78,1.07);				
	Multiplicative: ROR = 1.00 (0.87,1.15)				
**Secondhand smoke at home**					
Yes	Poor c	957 (21.1)	440 (30.9)	1.00	1.00
Yes	Fair	183 (4.0)	68 (4.8)	0.81 (0.60,1.09)	0.84 (0.62,1.15)
Yes	Good	661 (14.5)	228 (16.0)	0.75 (0.62,0.91)	0.77 (0.63,0.94)
No	Poor	1,454 (32.0)	381 (26.8)	0.57 (0.49,0.67)	0.66 (0.56,0.78)
No	Fair d	217 (4.8)	50 (3.5)	0.50 (0.36,0.70)	0.54 (0.38,0.76)
No	Good	1,071 (23.6)	257 (18.0)	0.52 (0.44,0.62)	0.64 (0.52,0.77)
Interaction b	Additive: RERI = −0.17 (−0.35,0.01); AP = −0.13 (−0.26,0.00);				
	S = 0.67 (0.47,0.95);				
	Multiplicative: ROR = 0.89 (0.78,1.02)				
**High-temperature cooking oil**					
Yes	Not good c	755 (16.6)	313 (22.0)	1.00	1.00
Yes	Good	594 (13.1)	200 (14.0)	0.79 (0.67,0.95)	0.82 (0.68,0.99)
No	Not good	1,863 (41.0)	574 (40.3)	0.58 (0.50,0.68)	0.78 (0.66,0.93)
No	Good d	1,331 (29.3)	337 (23.7)	0.54 (0.45,0.63)	0.67 (0.56,0.81)
Interaction b	Additive: RERI = 0.08 (−0.26,0.41); AP = 0.05 (−0.17,0.27);				
	S = 1.18 (0.54,2.62);				
	Multiplicative: ROR = 0.98 (0.75,1.28)				
**Coal used for cooking**					
Yes	Not good c	743 (16.4)	353 (24.8)	1.00	1.00
Yes	Good	483 (10.6)	203 (14.3)	0.89 (0.72,1.09)	0.89 (0.72,1.11)
No	Not good	1,875 (41.3)	534 (37.5)	0.60 (0.51,0.70)	0.74 (0.61,0.90)
No	Good d	1,442 (31.7)	334 (23.5)	0.49 (0.41,0.58)	0.62 (0.50,0.77)
Interaction b	Additive: RERI = −0.02 (−0.39,0.35); AP = −0.01 (−0.24,0.21);				
	S = 0.97 (0.54,1.73);				
	Multiplicative: ROR = 1.07 (0.82,1.39)				
**Solid fuel used for heating**					
Yes	Not good c	1,942 (42.7)	663 (46.6)	1.00	1.00
Yes	Good	1,363 (30.0)	413 (29.0)	0.89 (0.77,1.02)	0.94 (0.81,1.10)
No	Not good	662 (14.6)	210 (14.7)	0.93 (0.78,1.11)	0.85 (0.70,1.02)
No	Good d	576 (12.7)	138 (9.7)	0.70 (0.57,0.86)	0.68 (0.55,0.85)
Interaction b	Additive: RERI = −0.16 (−0.52,0.21); AP = −0.11 (−0.35,0.13);				
	S = 0.75 (0.43,1.29);				
	Multiplicative: ROR = 1.17 (0.87,1.57)				

a High-temperature cooking oil and coal used for cooking were included in kitchen ventilation, solid fuels used for heating was included in bedroom ventilation while ever smoking and secondhand smoke at home were included in both kitchen and bedroom ventilation.

b Adjusted for age (continuous), gender, education level (illiterate/primary school/middle school/high school/college), income 10 years ago (Yuan/year, continuous), body mass index (kg/m^2^, continuous), family history of lung cancer (yes/no), pack-years of smoking (continuous), ethanol consumption (ml/week, continuous), and study area (Dafeng/Ganyu).

c The joint effects category for further estimation of additive interaction.

d The reference category for measures of interaction on additive scale.

Table 4 presents the joint effects of household ventilation and the number of pollutant sources. Compared with living in a household with poor ventilation and three or four sources of indoor air pollutants, living in a household with good ventilation was associated with a decreased risk of lung cancer, in a dose-response manner relative to the number of pollutant sources (OR_adj_ = 0.72, 95% CI = 0.56, 0.94 for three or four pollutant sources; OR_adj_ = 0.54, 95% CI = 0.44, 0.67 for one or two pollutant sources; OR_adj_ = 0.24, 95% CI = 0.15, 0.38 for no pollutant source). An additive interaction was suggested between household ventilation and number of pollutant sources (RERI = −0.11, 95% CI: −0.24, 0.02; AP = −0.07, 95% CI: −0.14, 0.00); S = 0.85, 95% CI: 0.74, 0.98). Among individuals exposed to the same number of pollutant sources, those who lived in households with good ventilation were generally at a lower risk for lung cancer compared with those who lived in households with poor ventilation.

**Table 4:** Joint effects of household ventilation and number of pollutant sources on lung cancer risk.

Number of pollutant sources	Household ventilation	Control (%) (N = 4,543)	Case (%) (N = 1,424)	Crude OR (95%CI)	Adjusted OR (95%CI) a
3 or 4	Poor b	435 (9.6)	273 (19.2)	1.00	1.00
3 or 4	Fair	109 (2.4)	40 (2.8)	0.59 (0.40,0.87)	0.60 (0.40,0.91)
3 or 4	Good	313 (6.9)	137 (9.6)	0.70 (0.54,0.90)	0.72 (0.56,0.94)
1 or 2	Poor	1,781 (39.2)	517 (36.3)	0.46 (0.39,0.55)	0.57 (0.47,0.69)
1 or 2	Fair	272 (6.0)	74 (5.2)	0.43 (0.32,0.58)	0.52 (0.38,0.71)
1 or 2	Good	1,191 (26.2)	323 (22.7)	0.43 (0.36,0.53)	0.54 (0.44,0.67)
0	Poor	195 (4.3)	31 (2.2)	0.25 (0.17,0.38)	0.32 (0.21,0.48)
0	Fair	19 (0.4)	4 (0.3)	0.34 (0.11,1.00)	0.40 (0.13,1.23)
0	Good c	228 (5.0)	25 (1.8)	0.18 (0.11,0.27)	0.24 (0.15,0.38)
Interaction a	Additive: RERI = −0.11 (−0.24,0.02); AP = −0.07 (−0.14,0.00);				
	S = 0.85 (0.74,0.98);				
	Multiplicative: ROR = 0.93 (0.82,1.05)				

a Adjusted for age (continuous), gender, education level (illiterate/primary school/middle school/high school/college), income 10 years ago (Yuan/year, continuous), body mass index (kg/m2, continuous), family history of lung cancer (yes/no), pack-years of smoking (continuous), ethanol consumption (ml/week, continuous), and study area (Dafeng/Ganyu).

b The joint effects category for further estimation of additive interaction.

c The reference category for measures of interaction on additive scale.

## Discussion

In this large population-based case-control study, good ventilation in the kitchen and bedroom was inversely associated with lung cancer risk. Exposure to secondhand smoke at home, high-temperature cooking oil fumes, coal used for cooking or solid fuels used for heating were found to be positively associated with lung cancer. A strong dose-response pattern was observed between the number of pollutant sources and the risk of lung cancer. Additive interactions were observed between household ventilation and indoor air pollutants. Thus, our results suggest that adequate ventilation in household could reduce the exposure to multiple co-existing indoor air pollutants.

Our findings of an inverse association between good ventilation and lung cancer are consistent with the results reported in two previous studies [12], [13]. One hospital-based case-control study of 224 male and 92 female cases of lung cancer in Guangzhou Province, China, reported an increased lung cancer risk associated with no separate kitchen and poor air circulation in both males and females. The OR for lung cancer tended to decrease with increasing size of ventilation openings in living areas and kitchens [12]. Similarly, a population-based case-control study with 164 cases and 218 controls of female non-smokers in Taiyuan city, China, found that good ventilation conditions, such as multi-story houses and houses with more windows, separate kitchens, installed ventilators, and frequently open windows showed strong inverse associations with lung cancer risk [13]. However, these studies were not able to evaluate the potential effect modification of lung cancer risk between good ventilation and indoor air pollutants due to small sample sizes.

The indoor air pollutants included in our study were all associated with an increased lung cancer risk. However, the protective effects of household ventilation for lung cancer risk were different by the type of indoor air pollutants (Table 3). Secondhand smokers at home (OR_adj_ = 0.77) benefited most from good household ventilation, while exposure to high-temperature cooking oil fumes (OR_adj_ = 0.82) and active smokers (OR_adj_ = 0.85) also had benefited from good ventilation. Those exposed to coal used for cooking and solid fuels used for heating had borderline inverse associations with good ventilation. Smoking, especially secondhand smoke, is one of the major sources of indoor air pollutants in China [21]. Both smokers and non-smokers are often exposed to dense smoke in small and crowded kitchens and bedrooms without proper ventilation. At least 17 carcinogens are emitted in higher levels from sidestream smoke than from mainstream smoke, of which benzo(a)pyrene diol epoxide is directly associated with lung cancer [22]–[26]. Adequate ventilation both in the kitchen and in the bedroom could mitigate the positive association between lung cancer and smoking, including secondhand smoke.

Chinese home cooking often involves the use of cooking oil at high-temperature [27]. Consistent with the majority of epidemiologic studies [28]–[32], the present study also found a positive association between exposure to cooking oil fumes and the risk of lung cancer, particularly in Chinese women (OR_adj_ = 1.21, 95% CI: 1.03, 1.42, and OR_adj_ = 1.43, 95% CI: 1.12, 1.83 for men and women, respectively). Our study also found the positive association of exposure to high-temperature cooking oil fumes with lung cancer risk can be modified by good household ventilation.

Indoor emissions from household coal combustion contain high levels of carcinogenic polycyclic aromatic hydrocarbons (PAHs). Consistent with the findings in many previous studies and pooled analyses [33]–[38], the association between household coal combustion and lung cancer was confirmed in our study. Moreover, we further analyzed the relationship between years of coal used for cooking and lung cancer, a clear dose–response pattern was observed (*P*_trend_ <0.001). However, household ventilation was not observed to reduce the impact of coal used for cooking and solid fuels used for heating on lung cancer risk in the present analysis.

This study has certain limitations. First, as with most case-control studies, selection bias and recall bias may exist in our study. To minimize selection bias, all cases were recruited directly from the tumor registries while controls were selected from the local population databases. Since household ventilation conditions in the kitchen and bedroom are not well established protective factors for lung cancer, differential recall bias would have been minimal. However, our results might be conservative due to non-differential recall or interview bias, which might lead to observed associations biased toward the null. Second, the ventilation effect was collected by interview using an epidemiological questionnaire. Direct measurements might give more quantitative data, however it may also suffer misclassification bias because it can only represent the current exposure when the cases and controls were interviewed without considering the latent time for cancer development. Third, the data were not collected on the duration the participants lived in the same households and household renovations. Further analyses on the association of the duration with the risk of lung cancer development and when to ventilate were not possible. Fourth, outdoor air pollution may also affect indoor air quality, which in term may affect lung cancer risk. However, since both study sites are located in countryside, the less air-polluted areas. The potential impact of outdoor air pollution on indoor would be limited. Fifth, most of the cases were diagnosed at an advanced stage without surgical treatment, resulting in a relatively low participation rate (46.3%) and a low proportion of pathologic diagnosis (17%). Finally, uncontrolled confounding may have affected our results. In particular, we only examined the effects of air pollutants inside the household without considering environmental and occupational exposures outside the household, particularly for male workers. However, there were no clear differences between exposure variables and lung cancer among the subgroups after stratifying by ever smoking and gender, except for differences in CIs due to smaller sample size caused by stratification. Moreover, the lack of obvious differences between the crude ORs and the adjusted ORs indicate that the impact of potential confounding factors was limited. Despite these limitations, the present study is one of the largest population-based case-control studies to evaluate the association between household ventilation and lung cancer and the potential impact of good ventilation in reducing the exposure to indoor air pollutants as a preventive measure against lung cancer.

In conclusion, adequate ventilation in the household was observed to be inversely associated with lung cancer, most likely through the reduction of exposure to indoor air pollutants. The findings from this study suggest that household ventilation, in addition to tobacco cessation, should be considered as one of the public health measures for the prevention and control of lung cancer in the Chinese population.
